# Mental Health Problems and Educational Attainment in Adolescence: 9-Year Follow-Up of the TRAILS Study

**DOI:** 10.1371/journal.pone.0101751

**Published:** 2014-07-21

**Authors:** Karin Veldman, Ute Bültmann, Roy E. Stewart, Johan Ormel, Frank C. Verhulst, Sijmen A. Reijneveld

**Affiliations:** 1 University Medical Center Groningen, University of Groningen, Department of Health Sciences, Community & Occupational Medicine, Groningen, The Netherlands; 2 University Medical Center Groningen, University of Groningen, Interdisciplinary Center for Psychopathology and Emotion Regulation, Groningen, The Netherlands; 3 Erasmus Medical Center, Department of Child and Adolescent Psychiatry, Rotterdam, The Netherlands; Hôpital Robert Debré, France

## Abstract

**Background:**

This study examines if mental health problems at age 11 and changes in mental health problems between age 11 and 16 predict educational attainment of adolescents at age 19, overall and stratified by gender.

**Methods:**

Data from 1711 adolescents (76.8% from initial cohort) of the Tracking Adolescents' Individual Lives Survey (TRAILS), a Dutch prospective cohort study with 9year follow-up, were used. Mental health problems (externalizing, internalizing and attention problems) were measured by the Youth Self Report and the Child Behavior Checklist at ages 11 and 16. Difference scores for mental health problems between age 11 and 16 were calculated. Educational attainment was assessed at age 19.

**Results:**

Externalizing, internalizing and attention problems at age 11 were significantly associated with low educational attainment at age 19 (crude model). When adjusted for demographic variables and the other mental health problems, only the association for attention problems remained significant (odds ratio (OR), 95% confidence interval: 3.19, 2.11–4.83). Increasing externalizing problems between age 11 and 16 also predicted low educational attainment at age 19 (OR 3.12, 1.83–5.32). Among girls, increasing internalizing problems between age 11 and 16 predicted low educational attainment (OR 2.21, 1.25–3.94). For boys, no significant association was found for increasing internalizing problems and low educational attainment. For increasing attention problems between age 11 and 16 no significant association with low educational attainment was found.

**Conclusions:**

Externalizing, internalizing and attention problems at age 11 and an increase of these problems during adolescence predicted low educational attainment at age 19. Early treatment of these mental health problems may improve educational attainment, and reduce socioeconomic health differences in adulthood.

## Introduction

Successful educational attainment (e.g., achievement of at least upper secondary education) is associated with many favorable later-life social economic outcomes, including occupational achievement, financial security, or positive lifestyle behaviors. Mental health problems may negatively affect educational attainment and thus have adverse consequences during the entire life course [1]. The economic costs of childhood mental health problems are enormous. Smith and Smith [2] calculated that childhood mental health problems in the US cause a reduction in family income of about $10,400 per year. Mental health problems cover a broad range of emotional and behavioral problems, like externalizing problems (i.e. aggressive and rule-breaking behavior), internalizing problems (i.e. depressive and anxiety problems, withdrawn behavior, and somatic complaints) and attention problems.

Lee and colleagues [3], [4] found that mental disorders were associated with low educational attainment in nine high-income countries and seven middle- and low-income countries. For externalizing problems, consistent negative associations with educational attainment were demonstrated, e.g., negative associations have been found for conduct disorder and oppositional defiant disorder [5]–[8].

For the association of internalizing problems with educational attainment mixed results have been reported. While several studies reported that internalizing problems were not associated with educational attainment [6], [8]–[11], others showed associations with high school dropout, high school graduation failure and failure to enter college [7], [12]–[15]. For attention problems, several studies reported a statistically significant association with low educational attainment [5], [6], [8], [16].

To date, the evidence regarding the effects of mental health problems on educational attainment is inconclusive, probably due to methodological problems. First, although most studies use longitudinal data [5], [8], [11], [13]–[15], changes over time in mental health problems are seldom addressed [12], [16]. Second, measurement error may be relatively large due to reliance on just one informant. Even though it is known that use of multiple informants increases the reliability of the given information [17], [18], to our knowledge none of the aforementioned studies employed more than one informant, i.e. either the adolescent [8], [10] or a parent [9], [12]. Finally, most studies concern the US-setting and studies in the European setting are sparse [3], [4], [11]. When interpreting results from the US, differences between welfare systems have to be considered, i.e. results based on a liberal system cannot directly be translated to a social democratic system.

Thus, the aims of this study were to examine the prospective associations of mental health problems at age 11 and changes over time in mental health problems between age 11 and 16 on educational attainment at age 19. For both aims multiple informants were used, i.e. adolescents and their parents, and analyses were stratified by gender.

## Methods

### Ethics statement

The Dutch Central Committee on Research Involving Human Subjects approved all the TRAILS study protocols. All children and their parents provided written informed consent to participate.

### Study design and sample

TRAILS (TRacking Adolescents' Individual Lives Survey) is a prospective cohort study of Dutch adolescents, aiming to study the etiology and course of psychopathology [19], [20]. The study started in March 2001. Five municipalities in the Northern part of the Netherlands, both urban and rural areas, were asked to provide the name and address of all children born between October 1, 1989 and September 30, 1990 (first 2 municipalities) or October 1, 1990 and September 30, 1991 (last 3 municipalities). Of all children approached for participation (N = 3145), 6.7% were excluded because of mental or physical incapability or language problems. Of the participating group (N = 2934), N = 2230 children and one or both of their parents provided informed consent to participate (76%, mean age 11.09, SD 0.55). At baseline, no differences in psychopathology between responders and non-responders were observed [20]. Of the baseline participants (N = 2230), N = 2149 children participated in the second wave (96.4% of baseline, mean age 13.5, SD 0.53), N = 1816 in the third wave (81.4% of baseline, mean age 16.25, SD 0.69) and N = 1881 in the fourth wave (84.3% of baseline, mean age 19.05, SD 0.58). A more detailed description of the design, sample, procedures and non-response analysis can be found elsewhere [19]–[21]. The present study used data of 1711 (76.8% of baseline) TRAILS participants, from whom educational or occupational status was known at age 19.

### Measures

**Figure 1:**
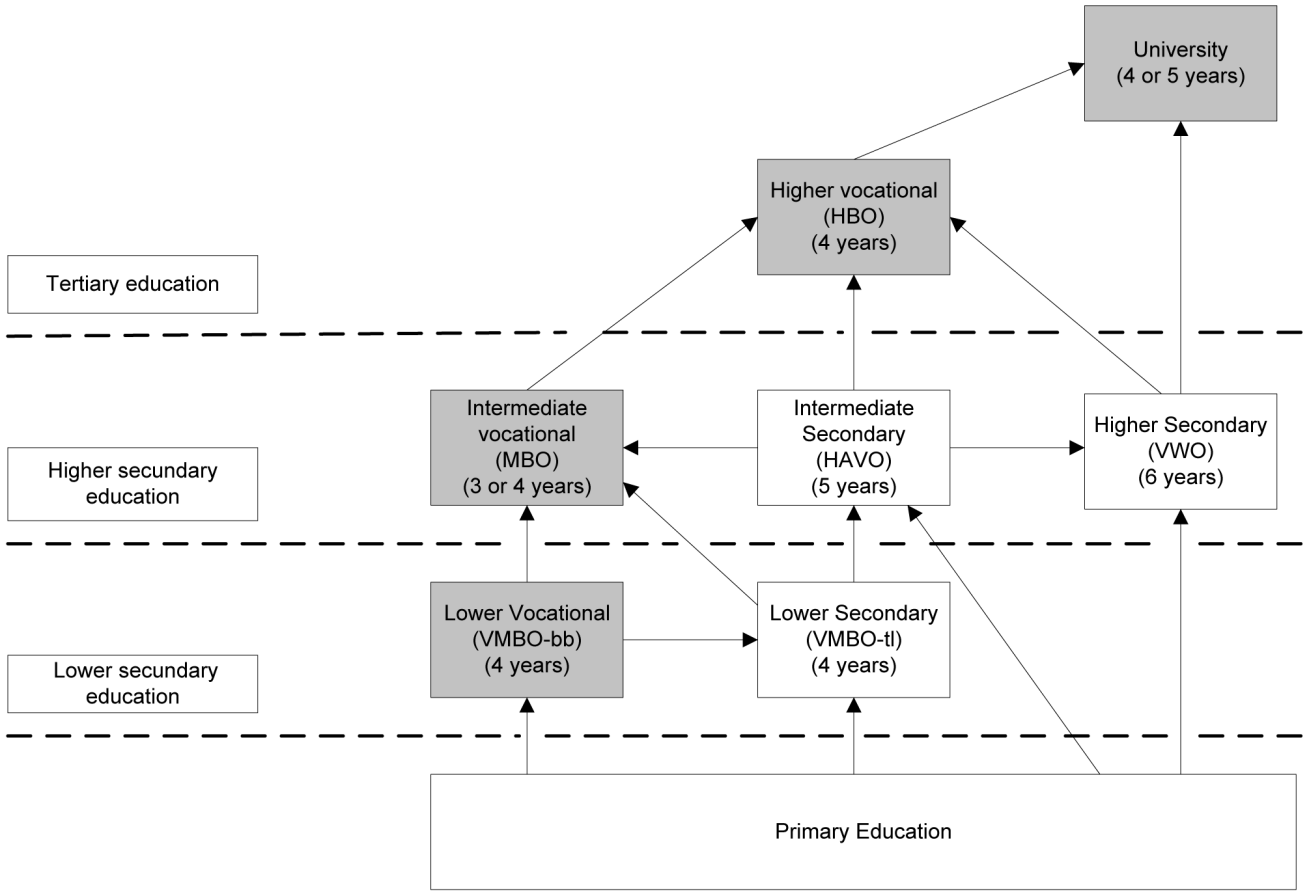
The Dutch educational system.

*Educational attainment* was measured at age 19 with two questions on the highest diploma obtained or on the current educational level if still at school. Educational attainment was categorized into: low (primary, lower vocational and lower secondary education), medium (intermediate vocational and intermediate secondary), and high (higher secondary, higher vocational and university) (see figure 1).

### The Dutch educational system

The Dutch educational system is presented in Figure 1. Children go to primary school from age 4 to 12. Admission to secondary school is based on the teachers' advice in primary school in combination with the outcome of a national test (CITO). Secondary education consists of a differentiated, multi-track system: schools that provide vocational education or job training and preparatory schools for entrance to tertiary education.

*Mental health problems* were measured using the Youth Self Report (YSR) and the Child Behavior Checklist (CBCL) at age 11 and 16 [22]. The YSR and CBCL contain a list of behavioral and emotional problems, scored as 0 =  not true, 1 =  somewhat or sometimes true, or 2 =  very or often true in the past 6 months. Standardized YSR and CBCL scores were combined into one measure, as multi-informant information provides a better prediction of mental health problems [17], [18]. The subscales aggressive behavior and delinquent behavior together form the scale externalizing problems (α = 0.85). Scale scores for externalizing problems range from 0 to 64, with higher scores indicating more externalizing problems. The scale internalizing problems contains the subscales anxious/depressed behavior, somatic complaints and withdrawn/depressed behavior (α = 0.87).). Scale scores range from 0 to 62, with higher scores indicating more internalizing problems. The subscale attention problems contains items regarding difficulties with concentrating, paying attention for a long time, daydreaming or getting lost in his/her thoughts. (α = 0.68). Scale scores range from 0 to 18, with higher scores indicating more attention problems.

*Intelligence* was determined at age 11 with the Vocabulary and Block Design subtests of the Revised Wechsler Intelligence Scales for Children (WISC-R) [23] leading to IQ-scores [24], [25].

*Academic performance* at age 11 was measured by asking the teacher five questions about the effort and achievement of their pupils, like “the pupil has a good work pace” and “the pupil performs beneath his standards”. Teachers rated the academic performance on a 5-point Likert scale. Some questions were recoded, and higher scores indicated better academic performance.

*Family composition* was measured at age 11 with two questions regarding the number of parents in the household and if biological parents were divorced in the period from birth to age 11.

*Parental educational level* was measured at age 11 with a question about the highest level of education of the father or mother. If both levels were known (which was the case in 85% of the parents), the highest level of education was used. Level of education was categorized into: low (primary, lower vocational and lower secondary education), medium (intermediate vocational and intermediate secondary and higher secondary education) and high (higher vocational education and university).

*Physical health* was measured at age 11 with the question: “How was your physical health the last two years?” Participants rated their health on a 5-point Likert-scale (1 =  very poor, 5 =  very good), which was recoded into 1 =  poor health (1, 2), 2 =  quite healthy (3) and 3 =  good health (4, 5).

### Data analyses

First, to test differences between low, medium and high educational groups, chi-quadrat-tests were performed for categorical variables (i.e. gender, parental educational level, family composition, physical health) and one-way ANOVA analyses for continuous variables (i.e. age, IQ, academic performance, externalizing, internalizing and attention problems).

Second, to examine whether externalizing, internalizing and attention problems at age 11 and 16 predicted educational attainment at age 19 multinomial logistic regression analyses were conducted. Multinomial logistic regression instead of ordinal logistic regression was performed because the test of parallel lines showed that the relationship between attention problems and the log-odds was not the same for all log-odds (X^2^ = 5.62, p-value = 0.02). Crude associations were calculated (model 1), followed by adjustments for age, gender, IQ, parental educational level and physical health (model 2). Model 3 was additionally adjusted for internalizing, externalizing and attention problems. Model 4 was additionally adjusted for academic performance (data not shown).

Third, to examine the relationship between changes over time in mental health problems and educational attainment, difference scores between age 11 and 16 were computed for externalizing, internalizing and attention problems. To test if the difference scores in externalizing, internalizing and attention problems between age 11 and 16 were associated with educational attainment at age 19, multinomial logistic regression analyses were performed. Model 1 was adjusted for baseline mental health problems, model 2 was additionally adjusted for age, gender, IQ, parental educational level and physical health. Model 3 was additionally adjusted for internalizing, externalizing and attention problems, and model 4 was additionally adjusted for academic performance (data of model 4 not shown).

Multiple imputation was performed because complete-case analyses could be conducted on only 1142 adolescents (66.7% of total sample). Data for internalizing, externalizing and attention problems were missing for 6.8% to 24.8% of respondents. Multiple imputation of missing data was performed on item level and thirty times to minimize the risk of bias and the loss of statistical power [26]. It was assumed that data are missing at random (MAR) or missing completely at random (MCAR) [27].

All analyses were conducted for the total sample and for boys and girls separately, using SPSS version 20.

## Results

### Sample characteristics

Of the total sample (N = 1711), 107 (6.2%) adolescents had a low level of educational attainment, 1211 (70.7%) a medium level of educational attainment, and 394 (23.0%) had a high level of educational attainment. Significant differences between educational groups were found for age, parental educational level, family composition, IQ, academic performance, physical health, externalizing problems (self- and parents' report), attention problems (self- and parents' report), difference score externalizing problems (self- and parents' report) and difference score internalizing problems (parents' report). Table 1 shows the sample characteristics per level of educational attainment.

**Table 1:** Descriptive information of the dependent, independent and confounding variables.

	Age	Educational attainment			P-value
Variables		Low	Medium	High	
Gender (N, %)	11				0.18
Boys		55 (51.4)	650 (53.7)	8.6)	
Girls		52 (48.6)	561 (46.3)	163 (41.4)	
Age (mean, SD)	19	19.2 (0.66)	19.0 (0.58)	19.1 (0.56)	0.01
Parental educational level (N, %)	11				<0.001
Low		47 (44.3)	279 (23.2)	6.4)	
Medium		45 (42.5)	486 (40.4)	17.6)	
High		14 (13.2)	438 (36.4)	392 (76)	
Family Composition (N, %)	11				<0.001
1 parent		37 (34.6)	262 (21.6)	60 (15.2)	
2 parents		70 (65.4)	949 (78.4)	334 (84.8)	
IQ (mean, SD)	11	87.9 (13.8)	96.5 (13.1)	110.8 (12.3)	<0.001
Academic Performance (mean, SD)	11	3.0 (0.8)	3.6 (0.8)	4.4 (0.5)	<0.001
Physical Health (N, %)	11				0.01
Poor		3 (2.8)	18 (1.5)	8 (2.0)	
Medium		11 (10.3)	106 (8.8)	23 (5.8)	
Good		93 (86.9)	1087 (89.8)	363 (92.1)	
Externalizing problems (mean, SD)	11				
Self-report (YSR)		10.2 (7.6)	8.9 (6.4)	7.8 (5.3)	<0.001
Parents' report (CBCL)		9.5 (6.9)	7.8 (6.4)	5.3 (4.6)	<0.001
Internalizing problems (mean, SD)	11				
Self-report (YSR)		13.0 (8.7)	11.7 (7.5)	11.2 (7.1)	0.09
Parents' report (CBCL)		8.3 (6.6)	7.9 (6.1)	7.3 (5.9)	0.15
Attention problems (mean, SD)	11				
Self-report (YSR)		4.9 (2.8)	4.5 (2.8)	3.9 (2.4)	<0.001
Parents' report (CBCL)		5.8 (3.8)	4.5 (3.3)	2.3 (2.1)	<0.001
Externalizing problems (mean change since 11, SD)	16				
Self-report (YSR)		3.0 (8.9)	1.5 (7.7)	0.3 (6.3)	<0.001
Parents' report (CBCL)		1.3 (7.2)	−1.6 (6.1)	−1.7 (4.4)	<0.001
Internalizing problems (mean change since 11, SD)	16				
Self-report (YSR)		1.1 (10.9)	−1.8 (8.3)	−2.0 (7.8)	0.71
Parents' report (CBCL)		0.3 (6.2)	−1.6 (6.0)	−2.2 (5.9)	0.01
Attention problems (mean change since 11, SD)	16				
Self-report (YSR)		1.2 (3.7)	0.9 (3.3)	0.8 (3.1)	0.52
Parents' report (CBCL)		−0.9 (3.1)	−0.9 (3.1)	−0.5 (2.1)	0.05

Chi-square tests are performed for categorical variables and one-way ANOVA analyses for continuous variables.

### Mental health at age 11 and educational attainment at age 19

For externalizing, internalizing and attention problems a significant association was found (model 1). After adjustment for age, gender, IQ, parental educational level and physical health, the association between internalizing problems and low educational attainment was no longer significant (model 2). Model 3 shows that after adjustment for the other mental health problems, only the association for attention problems at age 11 and low educational attainment remained statistically significant. When adjusted for academic performance, the association of attention problems at age 11 with low educational attainment at age 19 attenuated and was no longer significant (data not shown).

Results were the same for boys and girls (see Table 2).

**Table 2:** Multinomial logistic regression analysis of educational attainment at age 19 and externalizing, internalizing and attention problems at age 11.

	Model 1		Model 2		Model 3	
	OR	95% CI	OR	95% CI	OR	95% CI
*Low vs high educational attainment*						
Externalizing problems	2.33***	1.78–3.06	1.70**	1.18–2.46	1.41	0.94–2.11
Internalizing problems	1.36*	1.04–1.77	1.32	0.93–1.88	0.70	0.48–1.02
Attention problems	3.48***	2.60–4.66	1.78**	1.20–2.64	3.19***	2.11–4.83
*Middle vs high educational attainment*						
Externalizing problems	1.71***	1.44–2.03	1.56**	1.21–2.01	1.34*	1.03–1.74
Internalizing problems	1.14	0.98–1.32	1.25	1.00–1.56	0.75*	0.60–0.94
Attention problems	2.35***	1.97–2.81	1.53**	1.19–1.96	2.28***	1.77–2.95

*p<0.05; **p<0.01; ***p<0.001.

Model 1: Crude model | Model 2: Adjusted for age, gender, IQ, parental educational level and physical health |Model 3: Adjusted for age, gender, IQ, parental educational level, physical health, internalizing or/and externalizing or/and attention problems.

### Changes in mental health problems between age 11 and 16 and educational attainment at age 19

Table 3 shows that for increasing externalizing, internalizing and attention problems a significant association was found for the total sample, crude and adjusted for age, gender, IQ, parental educational level and physical health (model 1 and 2). When stratified by gender, the association between increasing internalizing problems and low educational attainment was statistically significant for girls only (OR 2.21, 1.25–3.94).

**Table 3:** Multinomial logistic regression analysis of educational attainment at age 19 and difference scores of externalizing, internalizing and attention problems between age 11 and 16.

	Model 1		Model 2		Model 3	
	OR	95% CI	OR	95% CI	OR	95% CI
*Low vs high educational attainment*						
Externalizing problems	3.12***	2.20–4.44	3.34***	2.12–5.28	3.12***	1.83–5.32
Internalizing problems	1.72**	1.20–2.46	1.52	0.92–2.52	0.71	0.42–1.19
Attention problems	2.30***	1.55–3.41	2.34***	1.42–3.86	1.51	0.85–2.68
*Middle vs high educational attainment*						
Externalizing problems	1.70***	1.38–2.10	1.92***	1.42–2.58	1.63**	1.18–2.23
Internalizing problems	1.21*	1.00–1.45	1.37*	1.04–1.80	0.74*	0.57–0.96
Attention problems	1.64***	1.34–2.02	1.95***	1.47–2.59	1.66**	1.22–2.24

*p<0.05; **p<0.01; ***p<0.001.

Model 1: Adjusted for baseline mental health problems | Model 2: Adjusted for age, gender, IQ, parental educational level, physical health, and baseline mental health problems | Model 3: Adjusted for age, gender, IQ, parental educational level, physical health, internalizing or/and externalizing or/and attention problems and baseline mental health problems.

After adjustment for the other mental health problems, no differences between boys and girls were found for the association between increasing externalizing, internalizing and attention problems and low educational attainment. For the total sample, only the association for increasing externalizing problems between age 11 and 16 and low educational attainment remained statistically significant (model 3).

## Discussion

The study examined the prospective associations of mental health problems and changes of mental health problems measured at age 11 and 16 with educational attainment of adolescents at age 19. We found that externalizing and attention problems at age 11 predicted low educational attainment at age 19. Moreover, increasing externalizing and internalizing (only for girls) between age 11 and 16 predicted low educational attainment at age 19.

Our finding of the prospective associations of externalizing and attention problems with low educational attainment is in line with previous US-based studies [5], [6], [8]. This indicates that externalizing and attention problems negatively affect school attainment, not only in a liberal welfare system, but also in a social-democratic welfare system as the Netherlands. It is possible that the underlying mechanism is similar for both contexts. One of the most likely mechanisms is social selection, i.e. that mental health problems cause low socioeconomic status. With the current data we were not able to address the social causation hypothesis, i.e. that low socioeconomic status cause mental health problems, but with future data of the TRAILS study it will be possible. Our finding of negative associations of externalizing and attention problems with low educational attainment might be due to the influence of externalizing and attention problems on other persons, such as teachers. Children and adolescents showing externalizing and attention problems are at greater risk to get involved with delinquent peers, to be dismissed from class and to receive negative feedback and therefore might have an increased risk of lower educational attainment [28].

After adjustment for internalizing and attention problems, the association between externalizing problems at age 11 and low educational attainment at age 19 was no longer significant. This might be explained by the fact that children with attention problems are at risk of having externalizing problems [29]. When looking at changes over time, McLeod and Fettes analyzed trajectories of externalizing and internalizing problems with educational attainment [12]. The findings of McLeod and Fettes regarding externalizing problems were similar with ours, i.e. increasing externalizing problems were significantly associated with low educational attainment.

For internalizing problems at age 11, the association with low educational attainment at age 19 attenuated and was no longer significant after adjustment for externalizing and attention problems. Prior research showed mixed findings, an explanation might be that the earlier reported associations were due to co-occurrence of internalizing problems with externalizing and attention problems. This is supported by the observation that studies which adjusted for psychiatric comorbidity, did not find significant associations [5], [6], [11], whereas studies which did not adjust for comorbidity found significant associations [7], [12]. In contrast, we found a significant associations of increasing internalizing problems between age 11 and 16 and low educational attainment at age 19. Girls with increasing internalizing problems have to be viewed as a high-risk group for low educational attainment, which needs specific attention. It can be speculated that girls enter a downward spiral, in which internalizing problems cause more problems at school, which in turn may lead to more internalizing problems.

The association between attention problems and low educational attainment was no longer significant after adjustment of academic performance. Probably both attention problems and academic performance are associated with educational attainment. Furthermore, attention problems and academic performance are related as well, i.e. children with attention problems are more likely to show poor academic performance. In the present study, the outcome was measured with a behavioral questionnaire. The scale attention problems contains mainly questions about attention problems and only a few questions about hyperactivity. Therefore, it was not possible to derive the diagnosis ADHD from this scale. However, several studies showed that childhood attention problems rather than hyperactivity predict educational attainment [16], [30], [31].

This study has several strengths and limitations. Data was used from a large, longitudinal representative population sample with 9-year follow up. With using repeated measurements we were able to examine mental health problems and their course over time from a life-course perspective. Another strength was the high retention rate at the different measurement waves, ranging from 81.4% to 96.4%. Moreover, we obtained information on adolescents' mental health problems from multiple informants, limiting the likelihood of information bias [17], [18]. We acknowledge that differences between the self- and parent-report of mental health problems disappeared, while these differences could have been informative as well. The level of mental health problems is based on self-report and parent-report and not on a diagnostic interview, the gold standard. However, in this study we were not only interested in the negative effects of severe mental health problems, but in the effects of mild mental health problems as well. In addition, reliable and valid measures were used to assess mental health problems (i.e. YSR and CBCL) [22].

Limitations concern results which are based on an imputed data set with the assumption that data was missing at random. If this assumption is not correct (e.g., that adolescents did not participate due to their mental health problems), it might have biased the results. We compared the imputed data with complete cases, but no differences were observed. Another point of attention is the calculation of difference scores of mental health problems between age 16 and 11. Therefore we were not able to examine if stable high mental health problems would have a negative effect on educational attainment. It would have been interesting to look at these problems as well, but due to the low number of adolescents with stable high mental health problems this was not possible [32].

Our study clearly showed that pre-adolescents' mental health problems and changes over time in mental health problems, are strongly associated with low educational attainment. This emphasizes the need for early detection, e.g., via well-child assessments and school monitoring [33]. Especially children whose mental health problems increase over time deserve attention, as they seem to be at greater risk of low educational attainment.

Early detection is of no use without early interventions, e.g., a better training of teachers and other school personnel to offer those children and their parents appropriate help, i.e. help that is tailored to meet the needs of children in their specific context. Behavioral problems and healthy behaviors are not separate entities, but are strongly related [34]. Therefore, interventions should focus on clusters of deviant behavior of children and adolescents at risk and require an integrated approach to improve the chances of these adolescents towards a successful entry of the labor market.
