# Correction: Mental Health Problems and Educational Attainment in Adolescence: 9-Year Follow-Up of the TRAILS Study

**DOI:** 10.1371/journal.pone.0115070

**Published:** 2014-12-02

**Authors:** The PLOS ONE Staff

There are errors in the numbers for Table 1. Please see the correct Table 1 here.

**Table 1:** Descriptive information of the dependent, independent and confounding variables.

	Age	Educational attainment			P-value
Variables		Low	Medium	High	
Gender (N, %)	11				0.18
Boys		55 (51.4)	650 (53.7)	(58.6)	
Girls		52 (48.6)	561 (46.3)	163 (41.4)	
Age (mean, SD)	19	19.2 (0.66)	19.0 (0.58)	19.1 (0.56)	0.01
Parental educational level (N, %)	11				<0.001
Low		47 (44.3)	(23.2)	25 (6.4)	
Medium		45 (42.5)	(40.4)	69 (17.6)	
High		14 (13.2)	438 (36.4)	392 (76)	
Family Composition (N, %)	11				<0.001
1 parent		37 (34.6)	262 (21.6)	60 (15.2)	
2 parents		70 (65.4)	949 (78.4)	334 (84.8)	
IQ (mean, SD)	11	87.9 (13.8)	96.5 (13.1)	110.8 (12.3)	<0.001
Academic Performance (mean, SD)	11	3.0 (0.8)	3.6 (0.8)	4.4 (0.5)	<0.001
Physical Health (N, %)	11				0.01
Poor		3 (2.8)	18 (1.5)	8 (2.0)	
Medium		11 (10.3)	106 (8.8)	23 (5.8)	
Good		93 (86.9)	1087 (89.8)	363 (92.1)	
Externalizing problems (mean, SD)	11				
Self-report (YSR)		10.2 (7.6)	8.9 (6.4)	7.8 (5.3)	<0.001
Parents’ report (CBCL)		9.5 (6.9)	7.8 (6.4)	5.3 (4.6)	<0.001
Internalizing problems (mean, SD)	11				
Self-report (YSR)		13.0 (8.7)	11.7 (7.5)	11.2 (7.1)	0.09
Parents’ report (CBCL)		8.3 (6.6)	7.9 (6.1)	7.3 (5.9)	0.15
Attention problems (mean, SD)	11				
Self-report (YSR)		4.9 (2.8)	4.5 (2.8)	3.9 (2.4)	<0.001
Parents’ report (CBCL)		5.8 (3.8)	4.5 (3.3)	2.3 (2.1)	<0.001
Externalizing problems (mean change since 11, SD)	16				
Self-report (YSR)		3.0 (8.9)	1.5 (7.7)	0.3 (6.3)	<0.001
Parents’ report (CBCL)		1.3 (7.2)	-1.6 (6.1)	-1.7 (4.4)	<0.001
Internalizing problems (mean change since 11, SD)	16				
Self-report (YSR)		1.1 (10.9)	-1.8 (8.3)	-2.0 (7.8)	0.71
Parents’ report (CBCL)		0.3 (6.2)	-1.6 (6.0)	-2.2 (5.9)	0.01
Attention problems (mean change since 11, SD)	16				
Self-report (YSR)		1.2 (3.7)	0.9 (3.3)	0.8 (3.1)	0.52
Parents’ report (CBCL)		-0.9 (3.1)	-0.9 (3.1)	-0.5 (2.1)	0.05

Chi-square tests are performed for categorical variables and one-way ANOVA analyses for continuous variables.
